# Mendelian randomization study reveals a causal relationship between coronary artery disease and cognitive impairment

**DOI:** 10.3389/fcvm.2023.1150432

**Published:** 2023-05-23

**Authors:** Shihan Xu, Yanfei Liu, Qing Wang, Fenglan Liu, Fengqin Xu, Yue Liu

**Affiliations:** ^1^ The Second Department of Geriatrics, Xiyuan Hospital, China Academy of Chinese Medical Sciences, Beijing, China; ^2^ National Clinical Research Center for TCM Cardiology, Xiyuan Hospital, China Academy of Chinese Medical Sciences, Beijing, China; ^3^ Graduate School of Guangdong Pharmaceutical University, Guangzhou, China

**Keywords:** causal effect, genome-wide association studies, mendelian randomization, cognitive impairment, coronary artery disease

## Abstract

**Background:**

Growing evidence suggests that Coronary artery disease (CAD) is associated with cognitive impairment. However, these results from observational studies was not entirely consistent, with some detecting no such association. And it is necessary to explore the causal relationship between CAD and cognitive impairment.

**Objective:**

We aimed to explore the potential causal relationship between CAD and cognitive impairment by using bidirectional two-sample mendelian randomization (MR) analyses.

**Methods:**

Instrument variants were extracted according to strict selection criteria. And we used publicly available summary-level GWAS data. Five different methods of MR [random-effect inverse-variance weighted (IVW), MR Egger, weighted median, weighted mode and Wald ratio] were used to explore the causal relationship between CAD and cognitive impairment.

**Results:**

There was little evidence to support a causal effect of CAD on cognitive impairment in the forward MR analysis. In the reverse MR analyses, We detect causal effects of fluid intelligence score (IVW: *β *= −0.12, 95% CI of −0.18 to −0.06, *P* = 6.8 × 10^−5^), cognitive performance (IVW: *β *= −0.18, 95% CI of −0.28 to −0.08, *P* = 5.8 × 10^−4^) and dementia with lewy bodies (IVW: OR = 1.07, 95% CI of 1.04–1.10, *P* = 1.1 × 10^−5^) on CAD.

**Conclusion:**

This MR analysis provides evidence of a causal association between cognitive impairment and CAD. Our findings highlight the importance of screening for coronary heart disease in patients of cognitive impairment, which might provide new insight into the prevention of CAD. Moreover, our study provides clues for risk factor identification and early prediction of CAD.

## 1. Introduction

CAD remains one of the leading causes of mortality worldwide, and the risk of CAD increases with age (1, 2). Simultaneously, cognitive impairment has become one of the greatest global challenges for public health (3). Growing evidence suggests that CAD is associated with cognitive impairment. The study by Xie et al. (4) found that incident CAD was signiﬁcantly associated with faster post-CAD-diagnosis cognitive decline by leveraging the longitudinal community-based data from the English Longitudinal Study of Aging. A 12-year follow-up in the Maastricht Aging Study (MAAS) reported that individuals with CAD show more cognitive decline in memory and processing speed than controls (5). However, these results from observational studies was not entirely consistent. Petrovitch H et al. (6) found that cognitive performance did not have significant association with ≥1 prior MI or history of coronary artery bypass graft surgery (CABG). And another study also found no significant correlation between CHD and more severe cognitive decline (7). CAD and cognitive impairment share many risk factors, such as elevated serum low-density lipoprotein cholesterol, high blood pressure, obesity, hyperglycemia and diabetes mellitus, and smoking (2, 3). The included individuals are often accompanied by these risk factors in observational studies, and the observational studies could not exclude the interference of these risk factors on the results. Therefore, it is unclear whether there is a causal relationship between CAD and cognitive impairment. This ambiguous relationship may confound some of the decisions made by clinicans, so it is necessary to explore the causal relationship between CAD and cognitive impairment.

**Figure 1:**
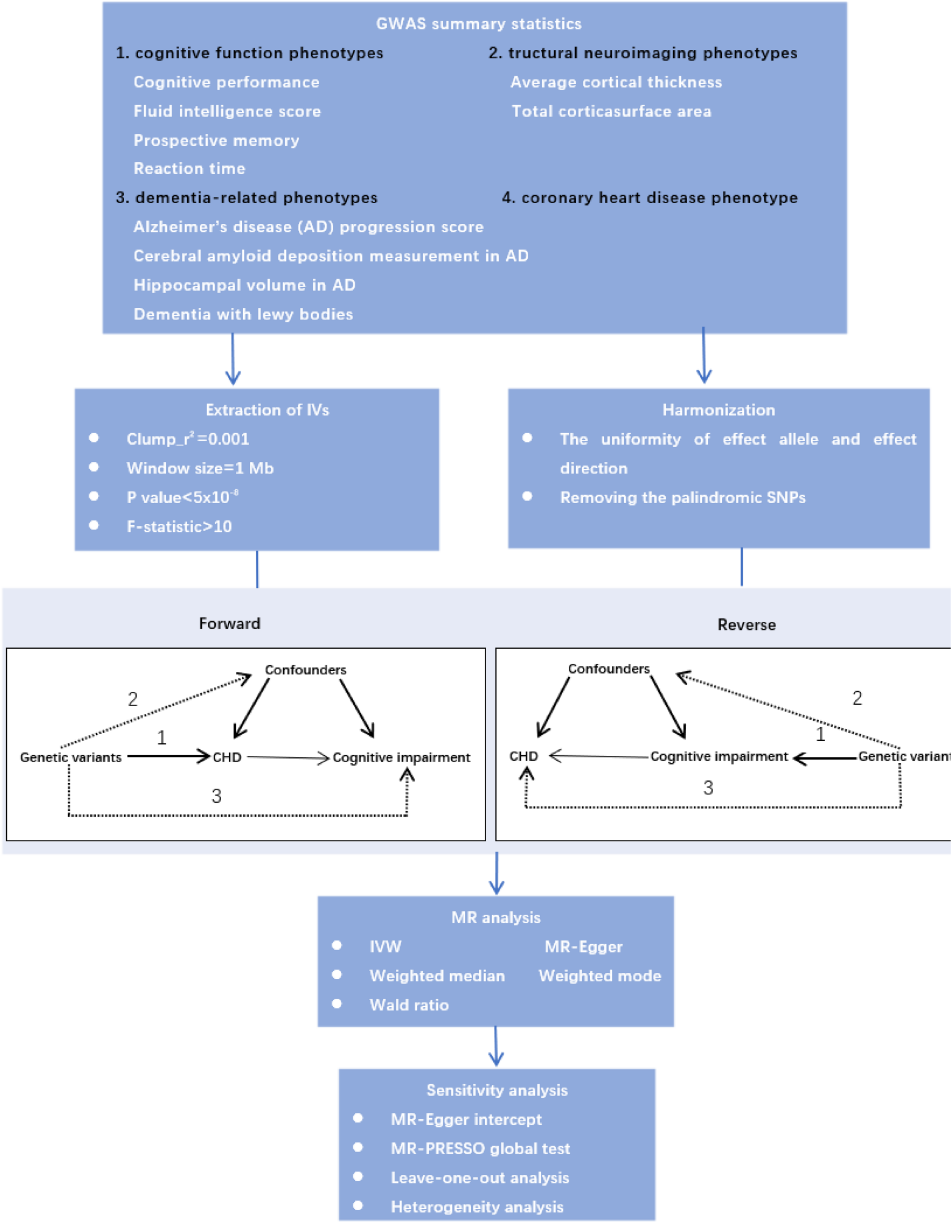
The flow chart of causal inference between CAD and cognitive impairment.

Mendelian randomization is a method that uses genetic variation associated with exposures to assess their possible causal relationship with outcomes, and it can reduce bias from confounding in epidemiological studies (8). Therefore, we performed bidirectional two-sample MR analyses to explore the causal relationship between CAD and cognitive impairment. Moreover, the underlying neurobiology of cognition is complex, we used detailed neurocognitive phenotypes, consisting of cognitive performance, fluid intelligence score, prospective memory, reaction time and structural neuroimaging phenotypes. In addition, cognitive impairment is a broad term that generally describes a decline in cognitive functions, and the severity of this impairment may range from mild symptoms to severe cognitive deficits that may be diagnosed as dementia. Therefore, we used the dementia-related phenotypes to explore the causal relationship between CAD and cognitive impairment from early to late stages. The study design is shown in Figure 1.

## 2. Materials and methods

### 2.1. GWAS summary data

We used publicly available summary-level GWAS data, and the detailed information is presented in Table 1. Summary-level data for CAD were obtained from the Coronary Artery Disease Genome-Wide Replication and Meta-analysis plus the Coronary Artery Disease (CARDIoGRAMplusC4D) Consortium cohort which assembled 60,801 cases and 123,504 controls from 48 studies (9). The data of cognitive performance from the Social Science Genetic Association Consortium (SSGAC) GWAS meta-analyzing published results combined a general cognitive ability study from the COGENT Consortium with a new genome-wide association analysis of cognitive performance in the UK Biobank (10). The data of fluid intelligence score and prospective memory were obtained from the Medical Research Council Integrated Epidemiology Unit (MRC-IEU) GWAS Pipeline (11). For reaction time, the summary-level GWAS data were extracted from Center for Cognitive Ageing and Cognitive Epidemiology (CCACE) analyzing the scores obtained on the reaction time test and genetic data of 330,069 UK Biobank participants (12). Average cortical thickness and total cortical surface area were included as structural neuroimaging phenotypes in the study, and the full GWAS summary statistic were extracted from a recent neuroimaging GWAS study including 51,665 participants (13). This study performed a genome-wide association meta-analysis of brain magnetic resonance imaging data to identify genetic variants affecting cortical structure. We also used dementia-related phenotypes, including Alzheimer's disease (AD) progression score, cerebral amyloid deposition measurement in AD, hippocampal volume in AD and lewy body dementia, the first three phenotypes from the genetic study of multimodal imaging Alzheimer's disease progression score (14), and the data of lewy body dementia from a recent study of GWAS associated with Lewy body dementia (15).

**Table 1:** Details of data source included in the study.

Phenotypes	Consortium	Sample Size	Population	Phenotype descriptions	MRC-IEU ID
CAD (9)	CARDIoGRAMplusC4D	184,305	European	The presence of myocardial infarction, acute coronary syndrome, chronic stable angina, or coronary stenosis >50%	ieu-a-7
Cognitive performance (10)	SSGAC	257,841	European	Meta-analysis of COGENT (first unrotated principal component of performance on three or more neuropsychological tests) and UKB (The standardized score on a test of verbal-numerical reasoning)	ebi-a-006572
Fluid intelligence score (11)	MRC-IEU	149,051	European	Fluid intelligence is the capacity to solve problems that require logic and reasoning ability, independent of acquired knowledge. And fluid intelligence score is a simple unweighted sum of the number of correct answers given to the 13 fluid intelligence questions. Participants who did not answer all of the questions within the allotted 2 min limit are scored as zero for each of the unattempted questions.	ukb-b-5238
Prospective memory (11)	MRC-IEU	152,605	European	Participants were asked to take a set of cognitive tests. Before the tests started, participants were instructed that at the end of the tests four coloured shapes will appear at the screen and despite they will be asked to touch the blue square they should touch the orange circle instead. This variable was analysed as an ordinal categorical variable, with lowest category reflecting better prospective memory test result and highest category reflecting worse prospective memory test result.	ukb-b-4282
Reaction time (12)	CCACE	360,069	European	In a test of 12 trials identifying identical cards, the first five were used as practice and the remaining seven were used for testing. The score is the mean time that participants correctly identify the matching cards in these seven trials.	NA
Total cortical surface area (13)	ENIGMA3	51,665	European	Global cortical surface area (total) measures extracted from *in vivo* whole-brain T1-weighted brain magnetic resonance imaging (MRI) scans in 34 regions and were quantified according to the Desikan-Killiany atlas. (mean = 169,647.43 ± 16,501.45 mm2)	NA
Average cortical thickness (13)	ENIGMA3	51,665	European	Average global cortical thickness measures extracted from *in vivo* whole-brain T1-weighted brain magnetic resonance imaging (MRI) scans in 34 regions and were quantified according to the Desikan-Killiany atlas. (mean = 2.45 ± 0.11 mm)	NA
Alzheimer's disease (AD) progression score (14)	ADNI	1,517	European	Novel multi-modal neuroimaging phenotype comprising imaging biomarkers of cortical amyloid burden and bilateral hippocampal volume - both imaging biomarkers used as input to disease progression modelling algorithm estimating biomarkers’ long-term evolution curves from population-based longitudinal data-algorithm computes shift in time required to optimally align a subjects’ biomarker trajectories with these population curves	NA
Hippocampal volume in AD (14)	ADNI	1,517	European	Bilateral hippocampal volume longitudinal measurements normalized by dividing by total intracranial volume	NA
Cerebral amyloid deposition measurement in AD (14)	ADNI	1,517	European	Ratio of standard uptake volumes ratio (SUVR) defined as SUV in the cortical region divided by the SUV in composite reference region comprising white matter, whole cerebellum, brainstem and pons -SUVR cut-off used to determine amyloid status equal to 0.7585	NA
Dementia with lewy bodies (15)	NA	6,618	European	Patients were diagnosed with pathologically definite or clinically probable lewy body dementia according to the fourth consensus report of the dementia with lewy body consortium	ebi-a-GCST90001390

### 2.2. Instrument variant (IV) selection

We extracted qualified IVs according to strict selection criteria. First of all, the SNPs with *P* < 5.00E-08 were extracted as candidate IVs. Secondly, the SNPs which were in linkage disequilibrium (*r*^2^ < 0.001, within a 10,000 kb window) would be excluded, and the SNPs were eliminated if they were palindromic with intermediate allele frequencies. Thirdly, the SNPs were excluded if they were not available in the outcome GWAS, and we did not use proxy SNPs in the study. In addition, we only used summary-level GWAS data from European ancestry to select the qualified IVs. Finally, we calculated the F statistics to further extract IVs strongly associated with phenotypes (F statistic >10), and these SNPs were considered valid and reliable IVs.

### 2.3. Mendelian randomization analysis

MR analysis must satisfy the following three assumptions: (1) the selected IVs must be strongly associated with the exposure; (2) the selected IVs should not be associated with potential confounders; (3) the selected IVs could only influence the outcomes through the exposure, but not other pathways. We chose five different methods of MR [random-effect inverse-variance weighted (IVW), MR Egger, weighted median, weighted mode and Wald ratio] to explore the causal relationship, and IVW was used as the major outcome, which is equivalent to fitting a weighted linear regression of the geneoutcome associations on the gene-exposure associations, with the intercept term constrained to zero (16). The estimated causal effects obtained from IVW method were compared with those obtained from MR-Egger regression, weighted median and weighted mode. MR-Egger regression allows for an unconstrained intercept term and provides a robust causal effect estimate, after adjusting for horizontal pleiotropy (17). The weighted median estimator can be considered an causal effect estimate without bias when up to 50% of the instruments are invalid, by estimating the causal effect as the median of the weighted ratio estimates (18). The weighted mode detects weaker causal effects than IVW, but presents less bias and lower type-I error rates (19). When the number of IVs extracted is less than 3, we used the Wald ratio to estimate the causal relationship.

### 2.4. Sensitivity analyses

We performed sensitivity analyses to verify the causalities obtained with bidirectional MR. First, the Cochran's *Q* tests were performed to detect the heterogeneity of the IVW method, and *P* < 0.05 in the Cochran's *Q* test indicated heterogeneity. Second, we performed the MR-egger intercept and the global MR pleiotropy residual sum and outlier (MR-PRESSO) to test and correct the potential horizontal pleiotropy of the selected IVs. The MR­Egger method was used to assess potential directional pleiotropy. A statistically significant intercept suggests directional pleiotropy, violating the instrumental variable assumptions (17). MR-PRESSO has three components which are detection of horizontal pleiotropy, correction for horizontal pleiotropy via outlier removal and testing of significant differences in the causal estimates before and after correction for outliers (20). Third, we conducted leave-one out analysis to explore the influence of each genetic variant on the outcome (21). All in all, the reliability of our causal effects were confirmed by the sensitivity analyses in both the forward and reverse MR results.

## 3. Results

A total of 611 SNPs were used as IVs for MR tests according to the selection criteria of IVs. And when average cortical thickness total corticasurface area, hippocampal volume in AD, AD progression score and cerebral amyloid deposition measurement in AD were used as exposures, the F values of the extracted SNPs were less than 10. Therefore, we did not performed reverse MR analysis when the above phenotypes were used as exposures. The full lists of IVs used for forward and reverse MR tests are provided in in Supplementary Table S1.

**Figure 2:**
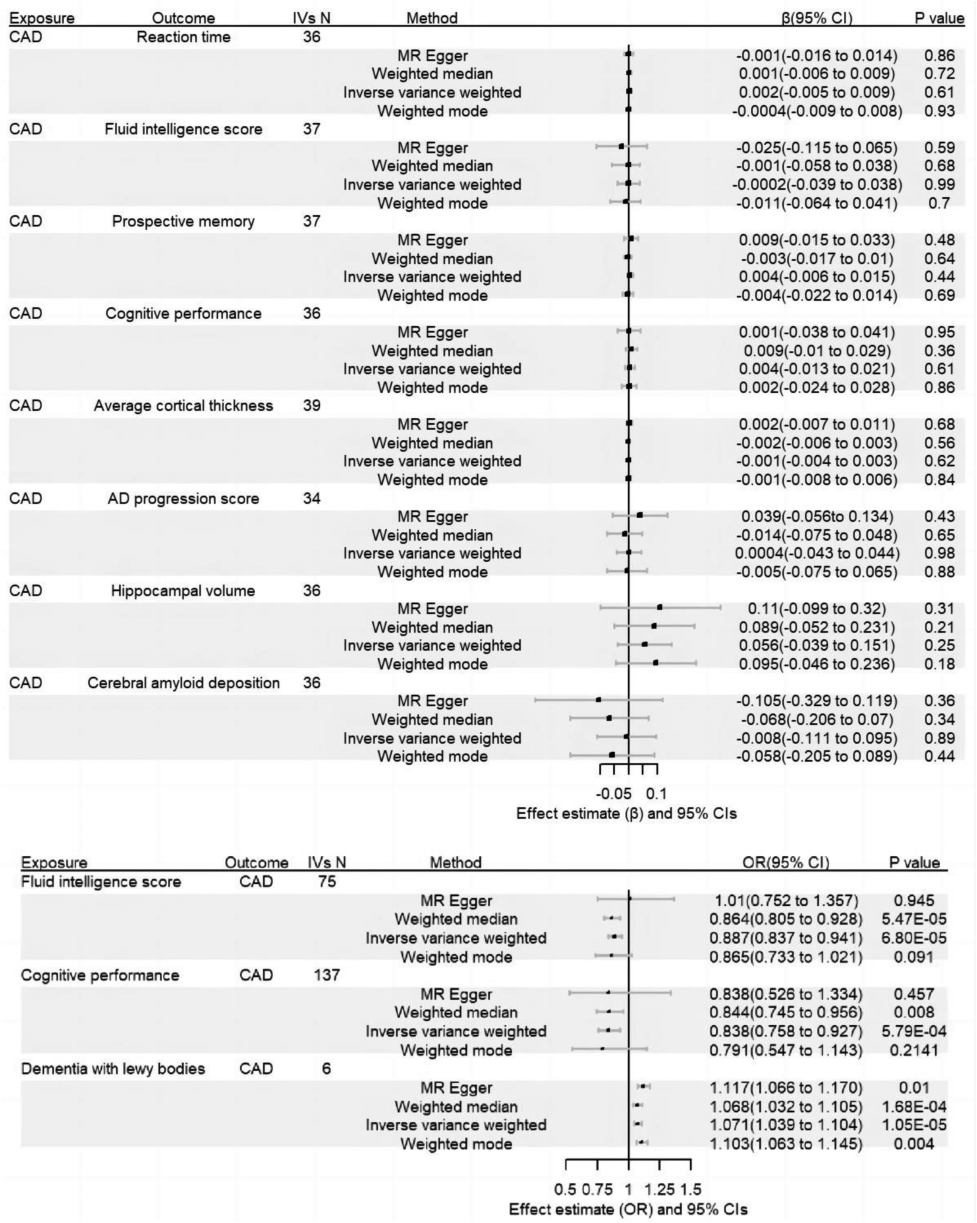
The result of bidirectional two-sample MR analyses.

According to the results of the forward MR anaylsis, there was little evidence to support a causal effect of CAD on cognitive impairment. Further, we failed to ﬁnd evidence that CAD impacted dementia-related outcome. In the reverse MR analyses, we detect causal effects of fluid intelligence score (IVW: *β *= −0.12, 95% CI of −0.18 to −0.06, *P* = 6.8 × 10^−5^), cognitive performance (IVW *β*=−0.18, 95% CI of −0.28 to −0.08, *P* = 5.8 × 10^−4^) and dementia with lewy bodies (IVW: OR = 1.07, 95% CI of 1.04–1.10, *P* = 1.1 × 10^−5^) on CAD. Complete results are presented in Supplementary Table S2. And the results were presented in Figure 2.

The sensitivity analyses confirmed the reliability of our result in both the forward and reverse MR. First of all, the MR-PRESSO test and the MR-egger intercept did not detect any evidence of horizontal pleiotropy. Second, Cochran's IVW *Q* test showed the *P*-values of these IVs from fluid intelligence score and cognitive performance were less than 0.05, but the results have less impact on causality because we chose a random-effect IVW. Third, leave-one-out analyses showed that no single SNP drove the causal estimates. Complete results of sensitivity analyses are presented in Supplementary Tables S3,S4.

## 4. Discussion

Observational studies had reported that CAD are associated with cognitive impairment. However it is undeniable that the results obtained from observational studies are largely influenced by confounding factors, such as hypertension (22), so it is difficult to distinguish whether the effect on cognition is caused by CAD or other factors. In the present study, we performed bidirectional two-sample MR analyses to systematically explore the causal relationship between CAD and cognitive impairment by controlling for confounding factors. And we did not identified CAD has a causal influence on cognitive impairment and dementia in the forward MR analysis. The clinical implication of these data is that the treatment of CAD may not directly protect against cognitive impairment. However, it is worth noting that the cardiovascular risk factors associate with cognitive impairment (23–25), the treatment strategies and lifestyle interventions currently recommended for CAD may indirectly have beneficial effects on the risk of cognitive impairment by improving these risk factors.

Moreover, we detect causal effects of fluid intelligence score, cognitive performance, and dementia with lewy bodies on CAD. Similar to our results, several observational studies also showed that cognitive impairment contributes to increased risk of coronary heart disease and in-hospital mortality (26, 27). However, these observational studies consistent with our results cannot completely exclude the influence of other confounding factors, so this result needs to be viewed with caution. The mechanism for the effect of cognitive impairment on CAD is still uncertain. A previous study showed that measuring blood levels of amyloid-beta 1–40 (Aβ40) were significantly and independently associated with arterial stiffness progression, incident subclinical atherosclerosis, and incident CAD (28). Stamatelopoulos et al. found that circulating Aβ40 is a predictor of mortality and improves risk stratification of patients with Non-ST-Segment Elevation Acute Coronary Syndrome (29). Amyloid-beta (Aβ) highly associated with cognitive impairment and dementia (30–33). The Aβ produced by the brain is able to cross the blood-brain barrier and thus enter the bloodstream (34–36), and then cause non-neurological amyloid lesions by depositing in distal organs and blood vessels (37, 38). Increasing evidence from animal studies have shown that Aβ plays a central role in vascular inflammation pathophysiology (39–43). It can promotes the secretion of cytokines, leading to oxidative stress, and activate a cascade of other proinflammatory responses, which ultimately lead to vascular disease (41, 44, 45). In summary, the causal relationship between cognitive impairment and CAD may be due to a series of pathological processes centered on Aβ. And further studies are needed to verify this association and to understand the mechanisms behind it.

The limitations of our study should be addressed. Frist, the small amount of data from ANDI may be underpowered enough to prevent potential relationships from being detected. When biometrics of AD from ANDI were used as exposures in the forward MR analysis, IVs could not be extracted, and this may be precisely due to insufficient data volume. Therefore, these analyses using biometrics of AD should be repeated when larger datasets become available. Second, we only analyzed the causal relationship between average cortical thickness and total corticasurface area and CAD. However different cortical regions have functional specializations (13), therefore, a more fine-grained analysis of cortical regions is needed to discover potential causal links. Third, the data we used were all from European ancestry, so our results may not be generalizable to populations of other ancestry. Finally, although IVs were obtained after strict screening criteria, caution is still needed. We performed the MR-egger intercept and MR-PRESSO to test and correct the potential horizontal pleiotropy of the selected IVs. However, there are limitations to theses approaches. Only if the gene pleiotropy is directional, and MR-egger intercept can detect the potential horizontal pleiotropy. In some cases, correction strategies of MR-PRESSO cannot completely eliminate the horizontal pleiotropy. Therefore, we look forward to better approaches to test and correct the potential horizontal pleiotropy of the IVs in the future.

In conclusion, we performed bidirectional two-sample MR analyses to systematically estimate the underlying causal relationships between CAD and cognitive impairment. We found some evidence to support a causal role of cognitive impairment in the pathogenesis of CAD. Future studies are needed to profoundly investigate the relationships between CAD and cognitive impairment, which might provide new insight into the prevention of CAD. Our findings highlight the importance of screening for coronary heart disease in patients of cognitive impairment. Moreover, our study provides clues for risk factor identification and early prediction of CAD.

## Data Availability

The original contributions presented in the study are included in the article/**Supplementary Material**, further inquiries can be directed to the corresponding authors.
